# Factors Associated With Satisfaction With Diabetes Care Among Patients Attending Public Diabetic Clinics in Dar es Salaam, Tanzania: A Cross‐Sectional Study

**DOI:** 10.1002/puh2.70002

**Published:** 2024-09-02

**Authors:** Emmanuel Z. Chona, Lusajo F. Kayange, Masunga K. Iseselo

**Affiliations:** ^1^ School of Nursing Muhimbili University of Health and Allied Sciences Dar es Salaam Tanzania; ^2^ Department of Clinical Nursing Muhimbili University of Health and Allied Sciences Dar es Salaam Tanzania

**Keywords:** diabetes mellitus, health services, patient satisfaction, public facilities, quality of health care

## Abstract

**Background:**

Diabetes is a major public health problem worldwide, currently affecting more than 537 million people. The disease is associated with high morbidity and mortality rates. Modern diabetes care has evolved toward more patient‐centered approaches, emphasizing individualized treatment plans and targets. This study sought to assess the level of satisfaction with diabetes care and related factors among patients attending public diabetes clinics in Dar es Salaam, Tanzania.

**Methods:**

This descriptive cross‐sectional study used a simple random sampling method to recruit 423 diabetic patients from May to October 2023. Data collection was conducted using a structured questionnaire administered by an interviewer. The collected data were coded and analyzed using Statistical Package for the Social Sciences (SPSS) version 25.

**Results:**

The mean (±SD) age of participants was 58.7 (±11.68) years. About half (51.1%) of participants reported being satisfied with the diabetes care provided, 26.2% were very satisfied, and 22.7% were dissatisfied. Participants who usually spend 1–3 h pursuing services at the facilities had 0.40 less odds of being dissatisfied with diabetes care offered at the clinics compared to those who spend more than 3 h on each attendance (adjusted odds ratio [adjusted OR] 0.40, 95% confidence interval [CI] 0.21–0.76, *p* = 0.005). For each one‐unit increase in the communication subscale score, the likelihood of satisfaction (as opposed to being very satisfied) increased (adjusted OR 1.23, 95% CI 1.09–1.38, *p* = 0.001). Each one‐unit increase in the accessibility subscale score increased the probability of satisfaction (adjusted OR 1.12, 95% CI 1.02–1.24, *p* = 0.023) and decreased the probability of dissatisfaction (adjusted OR 0.85, 95% CI 0.76–0.95, *p* = 0.004).

**Conclusion:**

These findings highlight the importance of effective communication and accessibility in improving patient satisfaction with diabetes care in public clinics. Health facilities and allied stakeholders should engage in continual capacity building among healthcare providers. Furthermore, other studies should be conducted at different levels of health facilities across the country to capture new insights on the satisfaction of homogenous sub‐groups of patients.

## Graphical abstract

Age group, duration since being diagnosed with diabetes, and time spent at the facility were strongly associated with patients’ satisfaction with diabetes care.

Communication and accessibility were aspects of care that showed a significant association with patients’ satisfaction with diabetes care.

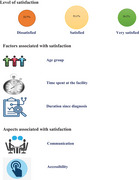

## 1 Introduction

Worldwide, the burden of diabetes is increasing rapidly due to the universal increase in the prevalence of obesity and lifestyle changes attributed to physical inactivity and unhealthy eating habits [1, 2]. About 6.7 million deaths due to diabetes were reported in 2021 [3]. By the year 2030, the total number of adult patients with diabetes mellitus is projected to rise up to 643 million [3]. In Africa, more than 24 million adults were living with diabetes in 2021. According to the IDF 2021 report, Tanzania had 2288,000 adults living with diabetes, making the prevalence of the disease to be 10.3% in the country [3].

Patient satisfaction is the extent to which patients are content with the health services they received from healthcare workers at a particular facility [4, 5]. It is an important factor in evaluating the quality of care, especially in treating chronic diseases such as diabetes mellitus [6, 7]. Despite reports that three‐quarters of adults with diabetes live in low‐ and middle‐income countries, healthcare delivery systems in those regions are poorly developed to accommodate the actual affected population [8, 9, 10]. Healthcare delivery systems in low‐ and middle‐income countries have received many negative comments, both from their clients and from society at large [11, 12]. These negative comments reflect the unsatisfactory quality of care offered by the health facilities geared by providers’ technical incompetence, limited accessibility, and unfavorable clinic environments [12, 13]. With these perturbing trends, studies on patient satisfaction have emerged as an increasingly crucial determinant on the ultimate quality of health services offered by the facilities [6, 7].

In resource‐constrained settings like Tanzania, where alternatives are unavailable for patients from lower socio‐economic backgrounds, patients continue seeking consultation in the same public diabetic clinics established within hospitals in district and regional capitals [14, 15]. However, comprehensive information on patient satisfaction with and factors influencing public diabetes care is still lacking. This highlights the need to carry out more studies to generate adequate evidence that will guide future interventions in the provision of quality diabetes care. This study sought to assess the level of satisfaction with diabetes care and related factors among patients attending public diabetes clinics in Dar es Salaam, Tanzania.

## 2 Materials and Methods

### 2.1 Study Design and Setting

A descriptive cross‐sectional study design was employed to assess satisfaction with diabetes care and associated factors among patients attending public diabetic clinics in Dar es Salaam, Tanzania. The study was conducted at Muhimbili National Hospital Mloganzila and the three municipal hospitals (Amana, Temeke, and Mwananyamala) in Dar es Salaam. As a business capital city, the region is administratively divided into five municipalities: Ilala, Kinondoni, Temeke, Ubungo, and Kigamboni. The clinics provide routine diabetic services, such as monitoring vitals, anthropometric measurements, glucose monitoring, medication prescriptions, dietary consultations, and health education sessions. Muhimbili National Hospital Mloganzila is the national referral hospital, and the diabetic clinic serves about 60–90 patients per day. The diabetic clinic at Amana serves an average of 40 patients per day. At Temeke, an average of 30 patients are consulted per day, and the diabetes clinics at Mwananyamala receive about 20 patients per day.

### 2.2 Study Population and Eligibility Criteria

This study involved a population of adult patients with diabetes (≥18 years) attending clinics at the selected public hospitals in Dar es Salaam, Tanzania. In this study, we excluded critically ill patients who were defined as having unstable vital signs, for example, diabetic ketoacidosis or hyperosmolar hyperglycemic state. Moreover, mentally unfit patients were excluded from the current study.

### 2.3 Sample Size and Sampling Procedure

We recruited 423 participants for this particular study. The sample size was calculated through the single‐population proportion formula [16] using 95% confidence interval (CI), 5% margin of error, 50% of patient satisfaction (as previous prevalence is not known), and a 10% nonresponse rate. Our sampling frame included patients receiving care at the public diabetic clinics. A stratified sampling approach was used to identify the diabetic clinics (strata), and a simple random sampling technique was employed to obtain the study participants at the diabetic clinics to attain the sample size required (423 participants). Participants were randomly approached by the research assistants at their consultation queue and invited to participate in the study. The proportional allocation method as proposed by Ajay and Micah [17] was used to determine the sample size at each clinic. Hence, this study recruited 193 diabetes patients at Muhimbili National Hospital Mloganzila, 77 diabetes patients at Amana, 102 diabetes patients at Temeke, and 51 diabetes patients at Mwananyamala.

### 2.4 Data Collection Tool

A structured interviewer‐administered questionnaire developed after a review of a previous related study [18] and modified to suit the specific objectives of this study was employed for gathering information from the study participants. The questionnaire consisted of two parts: Part I contained questions on the socio‐demographic characteristics of participants, and part II contained questions for the assessment of patients’ satisfaction with diabetes care and the factors associated with satisfaction. The questionnaire was developed in English and translated into Swahili, the national language, to enhance better understanding by the study participants.

### 2.5 Data Collection Procedure

Data were collected through an interviewer‐administered questionnaire by the principal investigators of this study with the assistance of four research assistants. They were trained before the data collection exercise to familiarize them with the study objectives, study methodology, data collection tool, and study protocols. Upon consultation by the research assistants, they were introduced to the nature and purpose of the study and provided consent before responding to the questions from the questionnaires. Each participant spent an average of 15–20 min responding to questions from the study tool.

### 2.6 Data Quality Control

As part of data quality control, the questionnaire was pretested in a pilot study done on 10% of the sample size who were finally excluded from the actual study sample. The pretesting aimed to check for the accuracy, consistency, and relevance of the questions. The questionnaire was revised and modified based on the responses to the pretest. A reliability test was performed by calculating the reliability coefficient (Cronbach's alpha), and its value was 0.759, which indicates a good reliability level for the tool with the studied sample [19].

### 2.7 Data Analysis

After data collection, the responses were coded and entered into the Statistical Package for the Social Sciences (SPSS) database program version 25.0 for analysis. Descriptive analyses were performed to present the socio‐demographic characteristics of the participants.

With regard to the patient's satisfaction questionnaire, there were five response categories (strongly agree, agree, uncertain, disagree, and strongly disagree) for all the scale items. The response categories were recorded as follows: favorable statements (strongly agree = 5, agree = 4, uncertain = 3, disagree = 2, strongly disagree = 1) and unfavorable statements (strongly agree = 1, agree = 2, uncertain = 3, disagree = 4, strongly disagree = 5); so that higher scores indicate greater satisfaction. An overall outcome variable assessing the level of patient satisfaction with diabetes care was designed on a three‐point ordinal scale from 3 (very satisfied), 2 (satisfied), and 1 (dissatisfied).

To calculate the subscale scores of the patient's satisfaction, the related variables were added to generate score‐based variables: technical expertise, time, communication, and accessibility. The higher scores indicated higher satisfaction. We expressed patient satisfaction scores as means and standard deviations. Variables with a *p* value <0.25 in the bivariate analyses were further analyzed in multivariate logistic regression to control the effect of confounders [20].

A multinomial logistic regression model was employed because the dependent variable had three outcomes (very satisfied, satisfied, and dissatisfied). Logistic regression models were calculated to show the association between (i) socio‐demographic characteristics and patient satisfaction and (ii) aspects of interaction (technical expertise, time, communication, and accessibility) and patient satisfaction. The results for the multinomial logistic regression analyses were presented as adjusted odds ratios (adjusted ORs) along with their respective 95% CIs, signifying precision. Variables with a *p* value <0.05 in the multinomial analysis were declared to be significantly associated with patients’ satisfaction with diabetes care.

## 3 Results

### 3.1 Socio‐Demographic Characteristics of Study Participants

The mean (±SD) age of participants was 58.7 (±11.68) years, with about three‐fourths (74.0%) of the participants in the age group of 42–65 years. In this study, more than half (52.5%) of participants were female patients, and the mean duration of diabetes since diagnosis was 8.98 (±4.17) years. At the time of the data collection, only 14.2% of participants had formal employment, and 42.8% of them had 6–12 months since their enrollment at their respective public diabetic clinics. The details of socio‐demographic characteristics of study participants are shown in Table 1.

**TABLE 1:** Socio‐demographic characteristics of patients who attended public diabetic clinics in Dar es Salaam, Tanzania, 2023.

Variables	Frequencies (*n*)	Percentages (%)
**Age of participant (years)**		
18–41	24	5.7
42–65	313	74.0
>65	86	20.3
**Sex of participant**		
Male	201	47.5
Female	222	52.5
**Duration of diabetes since diagnosis (years)**		
<6	114	27.0
6–10	124	29.3
>10	185	43.7
**Participant's marital status**		
Single	44	10.4
Married	155	36.6
Widow/Widower	157	37.1
Divorced	67	15.8
**Participant's level of education**		
No formal education	93	22.0
Primary education	140	33.1
Secondary education	127	30.0
College/Higher education	63	14.9
**Participant's occupation**		
Formally employed	60	14.2
Self‐employed	111	26.2
Unemployed	149	35.2
Retired	103	24.3
**Time since enrollment at the clinic (months)**		
<6	107	25.3
6–12	181	42.8
>12	135	31.9
**Average travel time from home to health facility by car (h)**		
<1	33	7.8
1–3	252	59.6
>3	138	32.6
**Average duration of time spent at the facility per visit (h)**		
<1	40	9.5
1–3	174	41.1
>3	209	49.4

### 3.2 Level of Patients’ Satisfaction With Diabetes Care

**FIGURE 1:**
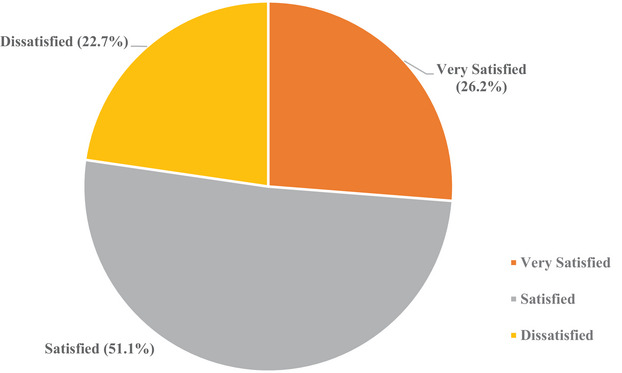
Level of satisfaction with diabetes care among patients who attended public diabetic clinics in Dar es Salaam, Tanzania, 2023.

Generally, about half (51.1%) of the participants were satisfied, 26.2% were very satisfied, and 22.7% were dissatisfied with diabetes care provided by the public diabetic clinics (Figure 1).

### 3.3 Association Between Socio‐Demographic Characteristics and Level of Patients’ Satisfaction With Diabetes Care

Upon multinomial logistic regression, participants of younger age (18–41 years) had 0.13 less odds of being satisfied with diabetes care provided at the clinics than the older group of more than 65 years (adjusted OR 0.13, 95% CI 0.04–0.42, *p* = 0.001); moreover, participants in the age group of 42–65 years had 0.35 less odds of being satisfied with diabetes care offered to them at the clinics than the older group of more than 65 years (adjusted OR 0.35, 95% CI 0.17–0.72, *p* = 0.004).

In terms of dissatisfaction, participants who usually spend 1–3 h pursuing services at the facilities had 0.40 less odds of being dissatisfied with diabetes care offered at the clinics compared to those who spend more than 3 h on each attendance (adjusted OR 0.40, 95% CI 0.21–0.76, *p* = 0.005). The details of the association between socio‐demographic characteristics and the level of patients’ satisfaction with diabetes care are shown in Table 2.

**TABLE 2:** Multinomial logistic regression analysis on socio‐demographics and level of satisfaction among patients who attended public diabetic clinics in Dar es Salaam, Tanzania, 2023.

	Level of patient satisfaction			
Variables	Satisfied adjusted OR (95% CI)	*p* value	Dissatisfied adjusted OR (95% CI)	*p* value
**Age group (years)**				
18–41	0.13 (0.04–0.42)	**0.001**	0.08 (0.01–0.49)	**0.006**
42–65	0.35 (0.17–0.72)	**0.004**	0.50 (0.22–1.12)	0.092
>65	1		1	
**Sex**				
Male	1.05 (0.63–1.76)	0.852	1.95 (1.06–3.57)	**0.031**
Female	1		1	
**Marital status**				
Single	5.67 (1.84–17.47)	**0.003**	3.48 (0.91–13.32)	0.068
Married	1.42 (0.69–2.95)	0.341	1.17 (0.49–2.77)	0.725
Widow/Widower	2.24 (0.99–5.04)	0.053	1.49 (0.58–3.80)	0.407
Divorced	1		1	
**Duration since diagnosis (years)**				
1–5	3.68 (2.04–6.61)	**<0.001**	1.71 (0.80–3.65)	0.164
6–10	14.98 (6.32–35.50)	**<0.001**	13.15 (5.20–33.25)	**<0.001**
>10	1		1	
**Travel time to facility (h)**				
<1	0.91 (0.35–2.38)	0.853	0.61 (0.19–1.95)	0.404
1–3	1.31 (0.74–2.31)	0.349	0.77 (0.41–1.47)	0.436
>3	1		1	
**Time spent at the facility (h)**				
<1	1.368 (0.57–3.30)	0.496	0.34 (0.09–1.24)	0.101
1–3	0.813 (0.47–1.39)	0.451	0.40 (0.21–0.76)	**0.005**
>3	1		1	

*Note:* Reference outcome: very satisfied; 1: reference category; bold elements shows significant statistical association at *p* < 0.05.

Abbreviations: CI: confidence interval; OR: odds ratio.

### 3.4 Association Between Aspects of Diabetes Care and Level of Patients’ Satisfaction

The mean (±SD) subscale scores of patient satisfaction were as follows: technical expertise (22.30 ± 1.98), time provision (10.22 ± 1.45), communication (16.64 ± 2.55), and accessibility (19.64 ± 2.70).

The multinomial logistic regression analysis demonstrated that with an increase in subscale scores by one unit, the likelihood of satisfaction increases in contrast to being very satisfied: communication (adjusted OR 1.23, 95% CI 1.09–1.38, *p* = 0.001) and accessibility (adjusted OR 1.12, 95% CI 1.02–1.24, *p* = 0.023). The details of the association between aspects of diabetes care and the level of patients’ satisfaction are shown in Table 3.

**TABLE 3:** Multinomial logistic regression for aspects of care associated with level of satisfaction among patients who attended public diabetic clinics in Dar es Salaam, Tanzania, 2023.

Level of satisfaction	Aspects of care	Adjusted odds ratio	95% CI	*p* value
Satisfied	Technical expertise	0.99	0.85–1.14	0.831
	Time	0.96	0.79–1.17	0.697
	Communication	1.23	1.09–1.38	**0.001**
	Accessibility	1.12	1.02–1.24	**0.023**
Dissatisfied	Technical expertise	1.00	0.85–1.17	0.982
	Time	1.03	0.83–1.28	0.772
	Communication	0.95	0.83–1.09	0.459
	Accessibility	0.85	0.76–0.95	**0.004**

*Note:* Reference category: very satisfied; bold elements shows significant statistical association at *p* < 0.05.

Abbreviation: CI: confidence interval.

## 4 Discussions

As observed from our results, about half (51.1%) of the participants were satisfied, 26.2% were very satisfied, and 22.7% were dissatisfied with the diabetes care provided by the public diabetic clinics. We found a statistical significant association between patients’ satisfaction and participants’ age group, sex, marital status, duration of diabetes, and time spent at the facility.

This study revealed that about half (51.1%) of the participants were satisfied, and 22.7% were dissatisfied with the diabetes care provided by the public diabetic clinics. The extent of dissatisfaction may be due to the individual variation in disease status and unavailability of some desired amenities at the health facilities [21]. These findings corroborate those of a study conducted among diabetic patients on follow‐up care at Tikur Anbessa Specialized Hospital in Addis Ababa, Ethiopia, where 54.3% of participants were satisfied with medical care services [10]. Inconsistent findings were reported by a study done in South India and Saudi Arabia among diabetes patients, where 70.1% and 87%, respectively, of the patients were satisfied with the healthcare services received [6, 22]. The discrepancy could be explained by socio‐economic status differences among the studied settings, financing mechanisms, and the priority given to quality of care by concerned agencies. Another study conducted in Pakistan to examine patient satisfaction with doctor–patient interactions revealed that 58% of the participants were very satisfied [18]. However, other factors related to the quality of care provided to diabetes patients at the facilities need further investigation.

In our study, participants of younger age (18–41 years) were less likely to be satisfied with the diabetes care provided at the clinics than the older group of more than 65 years. This association could be explained from different perspectives. First, the older people are usually more comfortable with paternalistic types of care rather than patient centered care. Second, this could be due to variations in perception of treatment services; the older people are more experienced with the care process and the potential weaknesses of the healthcare system [23, 24]. This finding is supported by a previous related study conducted in Pakistan, where the mean satisfaction level increased with an increase in age, and the maximum satisfaction level was found in older patients [25]. Another study done in Norway found a significant association between the age of participants and user satisfaction scores [26]. Inconsistent findings have been revealed by a previous related study conducted in Abha, Saudi Arabia, which reported the highest proportion of dissatisfaction with health services among participants aged 50–60 years compared to those of younger age groups [22]. Due to the limited number of studies and lack of consistent findings to support the association, this underscores the need for a more robust way of investigating the strength of the association on patients’ satisfaction across different age groups.

Male participants were 1.95 times more likely to be dissatisfied with the diabetes care provided at the clinics compared to female participants. One possible explanation for this finding is that, most of the female participants in the context of the current study were unemployed. This might enable them to have more time available to discuss disease management with healthcare providers during clinic consultation sessions [21, 27]. Hence, they are more likely to meet their expectations with the services provided at the clinics. The results are consistent with those found in a study done in Abha, Saudi Arabia, which reported that male participants expressed a significantly higher proportion of dissatisfaction with clinic services than females [22]. A previous related cross‐study conducted in Malaysia found that female participants were more satisfied with pharmaceutical care services provided in medication therapy adherence clinics [28].

Participants with less than 6 years since being diagnosed with diabetes in this study were 3.68 more likely to be satisfied with the diabetes care provided at the clinics than those with more than 10 years of life with diabetes. This finding corroborates those of a study conducted among Type 2 diabetes patients in Guangdong Province, China [29]. This study found that participants who usually spend 1–3 h pursuing services at the facilities had 0.40 less odds of being dissatisfied with the diabetes care offered at the clinics compared to those who spend more than 3 h on each visit. Lack of enough and well‐designed waiting places in proportion to the actual number of patients attending the clinic has been reported to annoy the clients, and that has reduced their chances of getting satisfied with the overall delivery of health services at the clinics [30, 31]. This finding has important implications for clinical practice as it provides contemporary insights into fundamental setbacks in the delivery of diabetes services that hinder intervention sustainability. In addition, the findings are suggestive of the inevitable capacity building of public health facilities through staffing improvements to accommodate the affected population in the respective catchment areas [32].

The findings of multinomial logistic regression analysis on aspects of care and level of satisfaction demonstrated that with an increase in subscale scores by one unit, the likelihood of satisfaction increases in contrast to being very satisfied: communication, and accessibility. This means that having good provider–patient communication and easy accessibility of public facilities was positively associated with patients’ satisfaction. The findings of this study are consistent with what was reported in a study conducted among diabetes mellitus patients in Pakistan to examine patient satisfaction with doctor–patient interactions [18]. It also highlights the need for a more organized system in the delivery of diabetes care at clinics to ensure that lessons learned can be of direct benefit in the management of diabetes in such resource‐constrained settings [14, 33]. These findings have practical implications for the provision of patient‐centered care and signify the need for health facilities and allied stakeholders to engage in continual capacity building among healthcare providers, with special emphasis on aspects of customer care to satisfy their consumers. However, having well‐designed and easily accessible diabetes treatment facilities in such resource‐constrained settings is problematic, and the inadequacy of clinics may contribute to multi‐sectoral inertia in intensifying diabetes care [15, 31].

### 4.1 Study Limitations

The current study was conducted at a national referral hospital and the three municipal‐level hospitals in Dar es Salaam. Hence, the findings from this study might underrepresent the actual situation of diabetes care at primary care–level facilities in the country because of differences in the availability of human and non‐human resources.

## 5 Conclusion

Patient satisfaction in public diabetic clinics is highly heterogeneous across various socio‐demographic features and aspects of diabetes care. The findings of this study highlight the importance of effective communication and accessibility in improving patient satisfaction with diabetes care in public clinics. Health facilities and allied stakeholders should engage in continual capacity building among healthcare providers. Furthermore, other related studies should be conducted regularly at different levels of health facilities across the country to capture new insights on the satisfaction of homogenous sub‐groups of patients.

## Author Contributions

**Emmanuel Z. Chona** and **Lusajo F. Kayange:** conceptualization, methodology, formal analysis, writing–original draft, writing–review and editing, project administration, validation. **Masunga K. Iseselo:** conceptualization, methodology, writing–review and editing, methodology, project administration, supervision, validation.

## Ethics Statement

We received ethical approval from the Institutional Review Board of Muhimbili University of Health and Allied Sciences (MUHAS‐IRB) with Ref. No. DA.282/298/01.C/1653.

## Consent

All study participants provided signed informed consent in the national language (Kiswahili) prior to data collection.

## Conflicts of Interest

The authors declare no conflicts of interest.

## Data Availability

The datasets for the current study are available from the corresponding author on request.
